# Plasma microRNA alterations between EGFR-activating mutational NSCLC patients with and without primary resistance to TKI

**DOI:** 10.18632/oncotarget.19874

**Published:** 2017-08-03

**Authors:** Yihan Ma, Xiaoyan Pan, Peiqi Xu, Yanjun Mi, Wenyi Wang, Xiaoting Wu, Qi He, Xinli Liu, Weiwei Tang, Han-Xiang An

**Affiliations:** ^1^Xiamen Cancer Hospital the First Affiliated Hospital of Xiamen University, 361003 Fujian, China; ^2^Department of Medical Oncology, Linyi Cancer Hospital, 276000 Shandong, China; ^3^Reproduction Center, The Second Affiliated Hospital of Kunming Medical University, 650101 Yunnan, China

**Keywords:** EGFR, EGFR-TKI, NSCLC, circulating miRNA, primary resistance

## Abstract

Epidermal growth factor receptor (EGFR) tyrosine kinase inhibitors (TKIs) have obtained excellent therapeutic effects against non-small cell lung cancer (NSCLC) harboring activating EGFR mutations. However, some patients have exhibited primary resistance which becomes a major obstacle in effective treatment of NSCLC. The mechanisms of EGFR-TKIs resistance involved are still poorly understood. Many studies suggest that miRNAs play an important role in regulating drug sensitivity of EGFR-TKIs. The aim of the present study was to examine differentially expressed miRNAs in plasma between EGFR-TKIs sensitive and EGFR-TKIs primary resistance patients. MiRNA microarray of plasma from patients’ blood identified 16 differentially expressed miRNAs of which 15 (hsv2-miR-H19, hsa-miR-744-5p, hsa-miR-3196, hsa-miR-3153, hsa-miR-4791, hsa-miR-4803, hsa-miR-4796-3p, hsa-miR-372-5p, hsa-miR-138-2-3p, hsa-miR-16-1-3p, hsa-miR-1469, hsa-miR-585-3p, ebv-miR-BART14-5p, hsa-miR-769-3p, hsa-miR-548aq-5p) were down regulated while only hsa-miR-503-3p was up regulated in primary resistant patients’ plasma. Volcano plot and hierarchical clustering were performed to examine the accuracy of the miRNAs. Then validation with quantitative real-time PCR was performed and the result was in accordance with the array data. Functional analysis of these differentially expressed miRNAs with Ingenuity Pathway Analysis (IPA) revealed a common signaling network including MYC, CCND1, IGF1 and RELA. In conclusion, our finding may play important role in understanding the mechanisms underlying the problem and should be further evaluated as potential biomarkers in primary resistance of NSCLC.

## INTRODUCTION

Lung cancer is the worldwide leading cause of cancer-related death [1]. It is classified into NSCLC and small cell lung cancer (SCLC) by histological category. More than 80 % of lung cancers are NSCLC, which includes adenocarcinoma, squamous cell carcinoma, large cell carcinoma, and bronchioalveolar carcinoma. Chemotherapy treatment and other treatment regime didn’t bring ideal effect and were far from satisfaction [2]. With recent developments of targeted therapy, EGFR -TKIs had achieved huge success and became one of the standard treatment-regimes of NSCLC [3]. It had been proved that the effectiveness of EGFR-TKIs was superior to chemotherapy in advanced NSCLC with activating EGFR mutations [4]. However, the efficiency of EGFR-TKIs is increasingly limited by drug resistance, approximately 10% of patients with EGFR-activating mutations do not exhibit objective responses to EGFR TKIs from the beginning (primary resistance). Although possible mechanisms like PTEN/PI3K/AKT pathway and NF-κB have been investigated in some studies, vital explanations are still missing [5].

MiRNAs are small (∼22-nucleotides long), non-coding, single-stranded RNAs that regulate gene expression post-transcriptionally. They are responsible for various biological and pathological processes including cancer development and progression [6]. In recent years, the dysregulation of miRNAs has been highlighted as one of the mechanisms underlying primary resistance of TKIs. It has been also proved that several miRNAs enhance therapeutic efficacy to EGFR-TKIs by modulating the sensitivity of cancer cells [7].

In our study, we compared plasma miRNA expressions between EGFR-TKI primary resistance patients and EGFR-TKIs sensitive patients. We hope that the profiles can provide a new perspective into the problem and further serve as a potential therapeutic approach to overcoming EGFR-TKIs primary resistance in NSCLC.

## RESULTS

### Differential miRNA expression profiles

**Figure 1:**
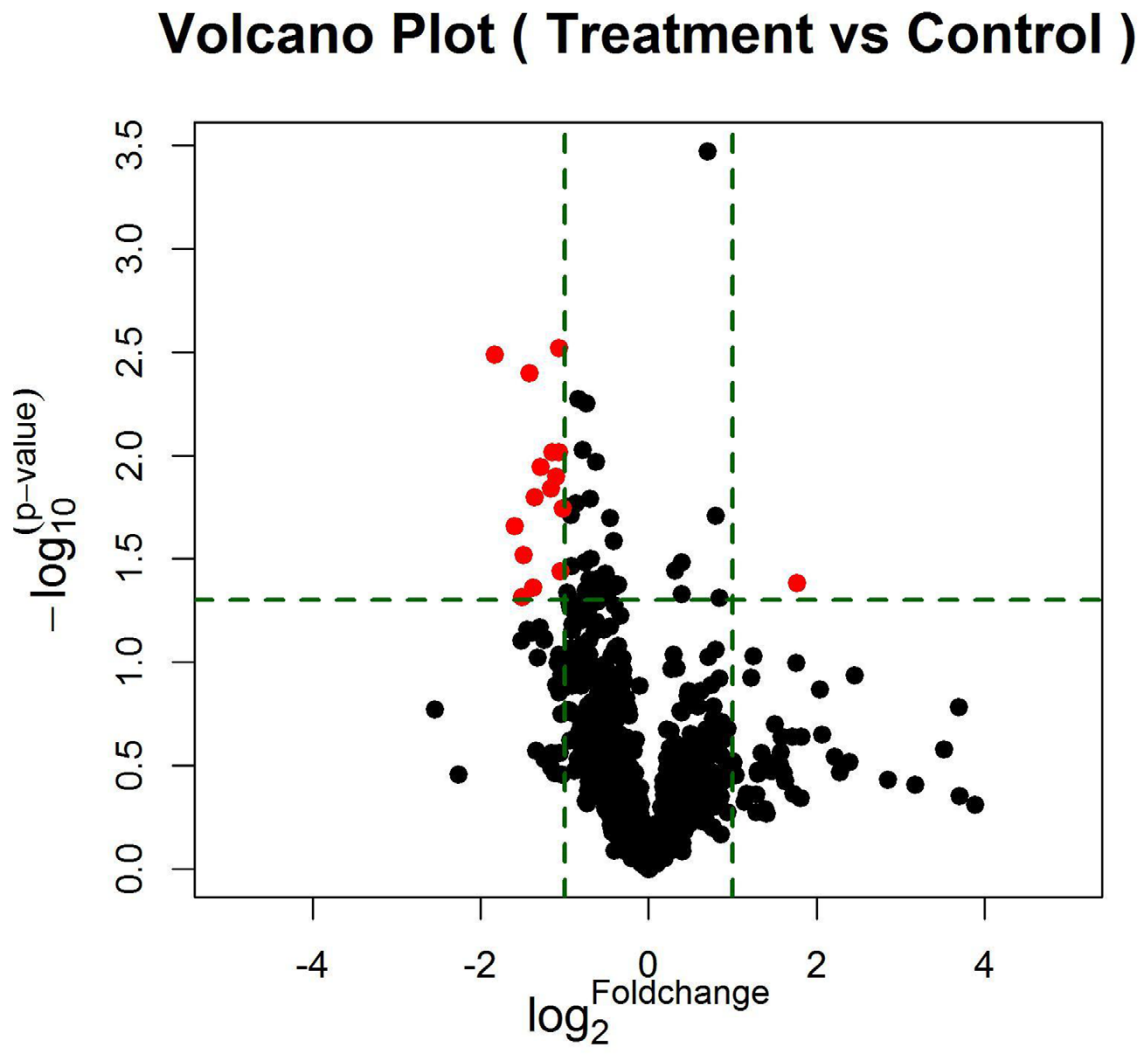
The vertical lines correspond to 2.0-fold up and down, respectively, and the horizontal line represents a p-value of 0.05 The red point in the plot represents the differentially expressed miRNAs with statistical significance. 16 miRNAs (red plots) passed the volcano plot filtering.

**Figure 2:**
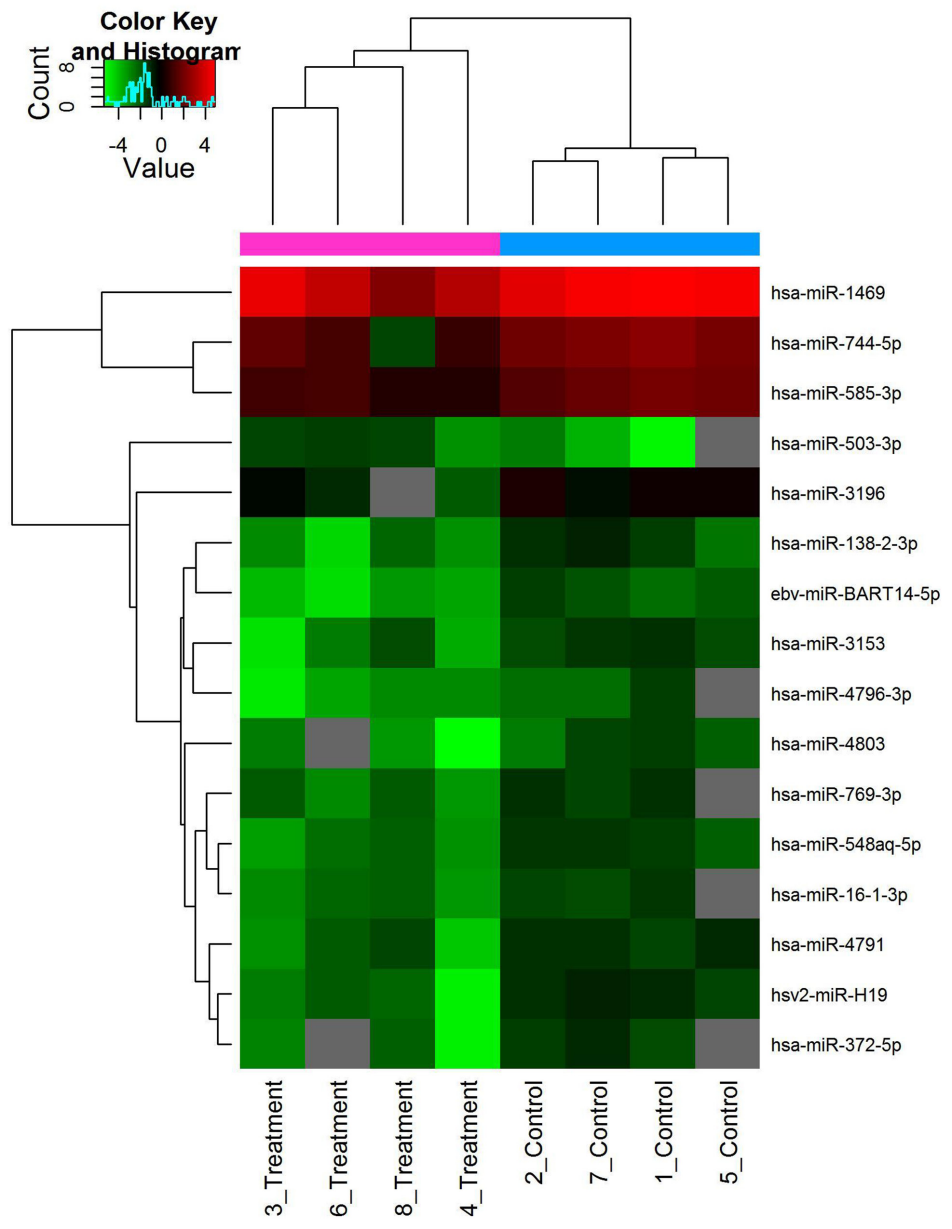
Hierarchical clustering of differentially expressed miRNAs Scale bar: up-regulated (red), down-regulated (green). Log2 transformed data were used.

According to data processing and analysis, specific miRNAs were identified to be differentially expressed between EGFR-TKI primary resistance patients and EGFR-TKI sensitive patients. The analysis revealed 16 miRNAs among which 15 miRNAs were down-regulated (hsv2-miR-H19, hsa-miR-744-5p, hsa-miR-3196, hsa-miR-3153, hsa-miR-4791, hsa-miR-4803, hsa-miR-4796-3p, hsa-miR-372-5p, hsa-miR-138-2-3p, hsa-miR-16-1-3p, hsa-miR-1469, hsa-miR-585-3p, ebv-miR-BART14-5p, hsa-miR-769-3p, hsa-miR-548aq-5p) while only hsa-miR-503-3p was up-regulated in EGFR-TKI primary resistance patients. Differentially expressed miRNAs were selected by volcano plot filtering (fold change ≥ 1.5 and P-value ≤ 0.05) as shown in Figure 1. There were 1 up-regulated miRNA and 15 down-regulated miRNAs as the fold numbers presented in Table 1. Then we performed hierarchical clustering of these miRNAs. We found these miRNAs clearly discriminated the EGFR-TKI primary resistance patients from EGFR-TKI sensitive patients, as shown in Figure 2.

**Table 1:** Differentially expressed miRNAs

Name	Fold change (resistance vs sensitivity)	P-value
*hsa-miR-503-3p*	*3.399*	*0.041*
*hsv2-miR-H19*	*0.374*	*0.004*
*hsa-miR-744-5p*	*0.410*	*0.011*
*hsa-miR-3196*	*0.482*	*0.036*
*hsa-miR-3153*	*0.389*	*0.016*
*hsa-miR-4791*	*0.446*	*0.014*
*hsa-miR-4803*	*0.351*	*0.048*
*hsa-miR-4796-3p*	*0.385*	*0.044*
*hsa-miR-372-5p*	*0.356*	*0.030*
*hsa-miR-138-2-3p*	*0.331*	*0.022*
*hsa-miR-1469*	*0.492*	*0.018*
*hsa-miR-585-3p*	*0.476*	*0.003*
*ebv-miR-BART14-5p*	*0.280*	*0.003*
*hsa-miR-769-3p*	*0.465*	*0.013*
*hsa-miR-548aq-5p*	*0.452*	*0.010*
*hsa-miR-16-1-3p*	*0.477*	*0.010*

### Validation with quantitative real-time PCR

**Figure 3:**
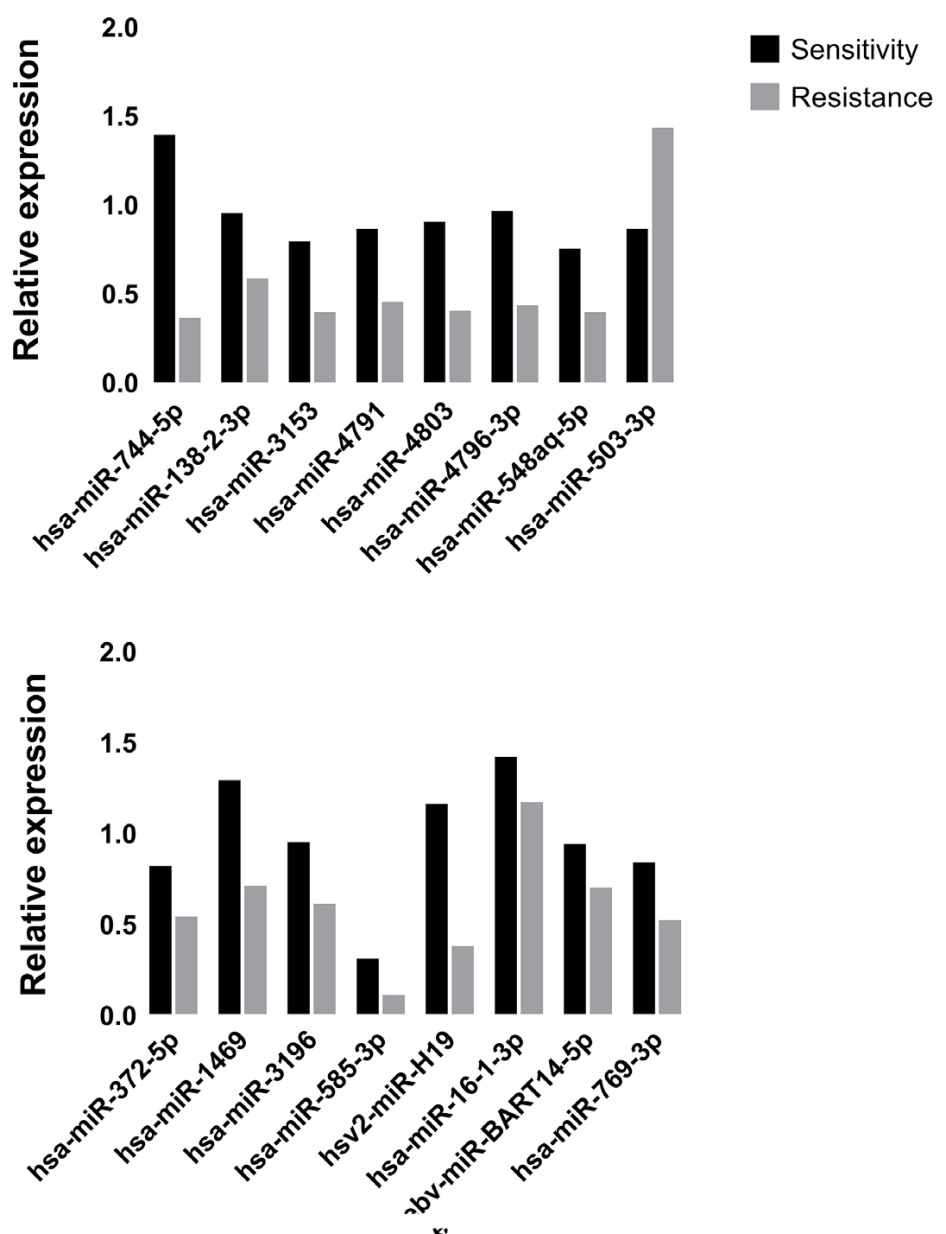
The expression of real-time qPCR was in accordance with microarray data

The expression levels of miRNAs were identified in 8 Plasma samples by RT-qPCR. As shown in Figure 3, Hsv2-miR-H19, hsa-miR-744-5p, hsa-miR-3196, hsa-miR-3153, hsa-miR-4791, hsa-miR-4803, hsa-miR-4796-3p, hsa-miR-372-5p, hsa-miR-138-2-3p, hsa-miR-16-1-3p, hsa-miR-1469, hsa-miR-585-3p, ebv-miR-BART14-5p, hsa-miR-769-3p, hsa-miR-548aq-5p were down regulated while only hsa-miR-503-3p was up regulated in primary resistant patients’ plasma.

### Network and molecular function analyses

**Figure 4:**
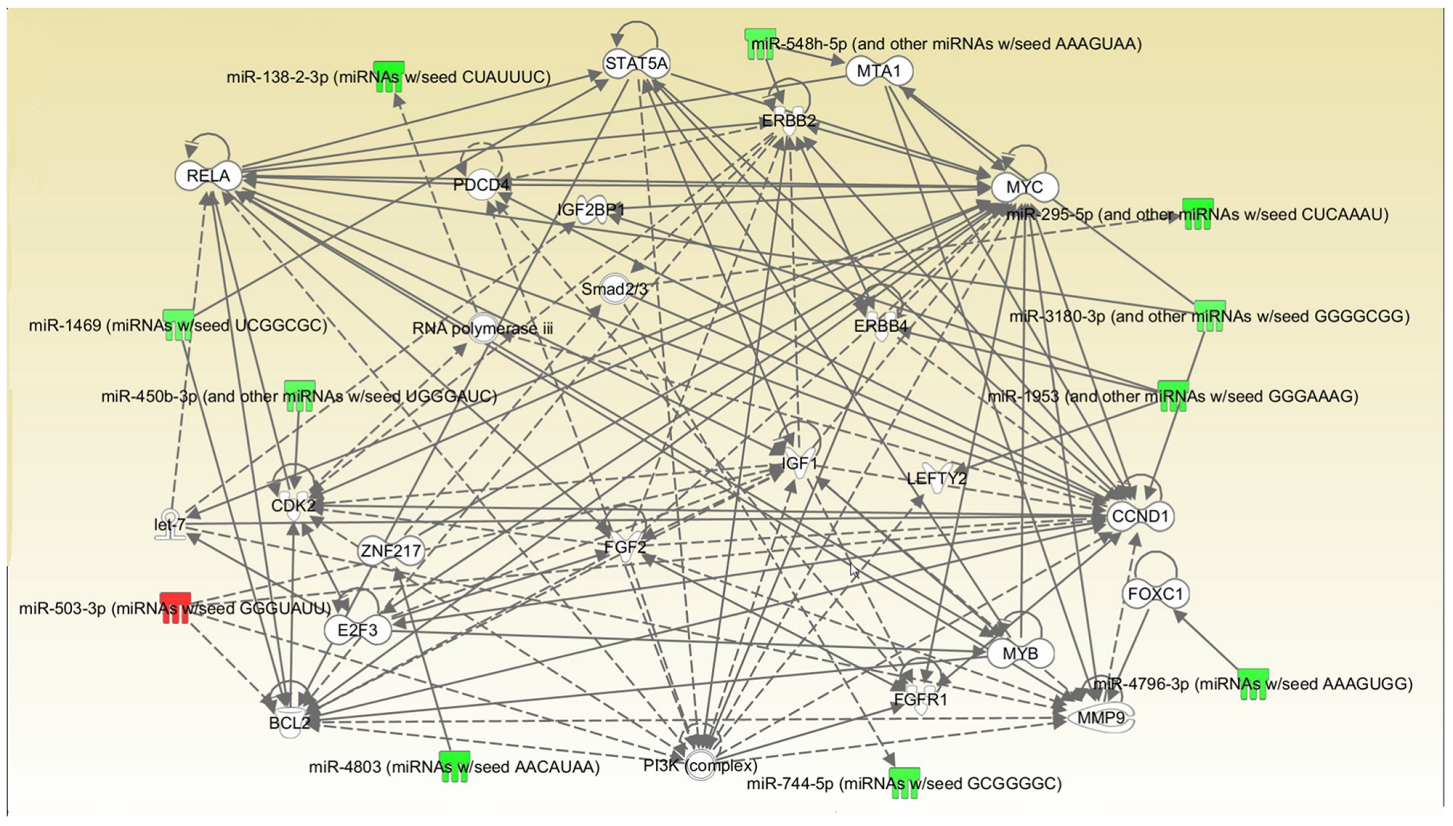
Interrelated networks of genes and miRNAs whose expression was different between TKI-sensitive patients and primary resistant ones One important network of interrelated miRNAs and target genes were identified. P=0.036.

16 miRNAs were mapped, network-eligible and classified into genetic network. 11 miRNAs at least share one overlapping gene in common as shown in Figure 4. IPA also depicted important biological pathway associated with this network. Meanwhile, different molecular events directly related to neoplasm were identified (i.e. cell death and survival, cellular development, cellular growth and proliferation). Furthermore, their relationships with diseases and disorders were also assessed.

The other 5 miRNAs were detected in separate networks without overlapping due to the lack of commonly-shared gene.

## DISCUSSION

To investigate the possible mechanisms underlying primary resistance to EGFR-TKIs, we identified patients exhibiting primary resistance with NSCLC harboring EGFR activating mutations. We found several miRNA alterations in plasma related to primary resistance in a small subset of patients. The study well represented the demographic and clinicopathologic characteristics of patients with EGFR-mutant NSCLC, which could provide practical considerations for EGFR-TKIs treatment in the precision treatment era.

Our results show that a total of 16 plasma miRNAs were differentially expressed including miR-744-5p, miR-138-2-5p, miR-3153, miR-4791, miR-4803, miR-4796-3p, miR-372-5p, miR-548aq-5p and hsv2-miR-H19*.* These miRNAs may play an important role in primary resistance to NSCLC with activating EGFR mutations. In addition, one network was detected and emerged via overlapping genes with the analysis of IPA concerning cell death and survival. Recent studies have reported on the role of miRNAs in EGFR-TKIs resistance. MiR-21 was verified to have significantly higher expression in resistant patients [8, 9]. Our findings are in agreement with these papers that miRNAs play and important role in the mechanism underlying EGFR-TKIs resistance. Furthermore, we provided more new miRNAs which could possibly add new knowledge to the subject.

MYC exhibits extremely important functions in cell cycle progression, apoptosis and cellular transformation. Functional experiment showed that miR-744 suppresses tumor by targeting c-Myc which leads to the inhibition of HCC cell growth [10]. What’s more, it has a more specific relationship with lung cancer and has been proved to take part in the enhanced glutamine metabolism which could possibly be a surrogate marker to predict the respond to EGFR-TKIs [11]. Its emergence here probably indicates the potential to be a biomarker or target. CCND1 is reported to be a key target in NSCLC development and can be regulated by many different factors like mir-146a-5p and mir-134 PSAT1 [12-14]. Relevant mechanisms include causing cell cycle arrest at the G0/G1 phase and great difference is shown in inhibiting cell proliferation, migration, invasion, and promoting apoptosis by targeting CCND1. Besides, CCND1 has a close relationship with MYC so the function they perform together in this network may worth investigating. RELA stands for NF-κB, a present in almost all cell types and is the endpoint of a series of signal transduction events such as cell growth, tumorigenesis and apoptosis. Recently a study showed that NF-κB activation may replace the oncogenic EGFR signaling in NSCLC when EGFR is inhibited by TKI in the presence of the T790M [15]. Therefore, its inhibition may be a promising therapeutic option for those who progress after original EGFR-TKIs treatment. PI3K is originally involved in normal cellular functions which in turn involved in cancer. However, recently a growing body of evidence has shown that it could do more in NSCLC and TKI resistance especially take PTEN/PI3K/AKT pathway into consideration [16]. Since clinical PI3K inhibition has been somewhat disappointing, people are now trying to co-inhibit PI3K with other factors at the same time to achieve a better outcome [17, 18]. MMP9 is closely associated with cancer, due to its role in extracellular matrix remodeling and angiogenesis. Although there are some paper mentioning its role in lung cancer, its place in TKI resistance remains largely unknown [19]. BCL2 and ERBB2 are mostly referred to in NSCLC but not TKI treatment. For example, BCL2 has been proved to be connected with resistance to platinum-based chemotherapy while ERBB2 is merely said to be involved in the tumorgenesis [20]. So, whether they could serve as potential target in overcoming primary resistance is still to be uncovered.

In conclusion, the emergence of key regulators such as MYC, CCND1 and RELA could be important in distinguishing the relevant mechanisms. Genetic mutation or deletion leads to the inactivation of tumor suppressor genes in the carcinogenesis. Furthermore, miRNAs modify tumor suppressor genes at transcriptional level and suppress their functions in response of TKI [21].

Surely the problem is still from fully understood. For example, ethnicity, T790M and other bypass mechanisms. Therefore, future studies are needed to examine these in detail and will provide insight into their roles in NSCLC TKI treatment.

## MATERIALS AND METHODS

### Patients and samples collection

Blood samples were collected from NSCLC patients with EGFR-activating mutations who received gefitinib or erlotinib between 2013 and 2015 treated in the Xiamen Cancer Hospital, the First Affiliated Hospital of Xiamen University. Blood samples were obtained with patients’ informed consent. EGFR activating mutations were defined as mutations known to be associated with EGFR TKI sensitivity, including exon 19 deletion and L858R. Imaging data were reviewed to evaluate treatment responses according to the Response Evaluation Criteria in Solid Tumors recommended by the WHO, defined as complete remission (CR), partial remission (PR), stable disease (SD), and progressive disease (PD) on the basis of the published literature. We defined primary resistance as disease progression in <3 months (90 days) without any evidence of objective response while receiving EGFR TKI treatment. For each sample, 5–10mL of blood was taken in EDTA-containing vacuum tubes. Plasma and blood cells were separated by centrifugation at 820 × *g* for 10 min at 4°C, Plasma was then centrifuged at 2500 × *g* for 10 min at 4°C. Plasma was stored at −80°C.

### RNA extraction

Plasma was separated within half an hour with two centrifuging steps (850g/10min and 2400g/10min) at 4°C. Total RNA was isolated from 200μl plasma. Total RNA was isolated using TRIzol (Invitrogen) and purified with RNeasy mini kit (QIAGEN) according to manufacturer’s instructions. RNA quality and quantity was measured by using nanodrop spectrophotometer (ND-1000, Nanodrop Technologies) and RNA Integrity was determined by gel electrophoresis.

### MiRNA labeling and array hybridization

RNA labeling and array hybridization was according to Exiqon’s manual. After quality control, the miRCURY™ Hy3™/Hy5™ Power labeling kit (Exiqon, Vedbaek, Denmark) was used according to the manufacturer’s guideline for miRNA labelling by following steps: 1μLRNA in 2.0 μL of water was combined with 1.0 μL of CIP buffer and CIP (Exiqon). The mixture was incubated for 30 min at 37°C. The Reaction was terminated by incubation for 5 min at 95°C. Then 3.0 μL of labeling buffer, 1.5 μL of fluorescent label (Hy3TM), 2.0 μL of DMSO, 2.0 μL of labeling enzyme were added into the mixture. The labeling reaction was incubated for 1 h at 16°CTerminated by incubation for 15 min at 65°C. After stopping the labeling procedure, the Hy3™-labeled samples were hybridized on the miRCURYTM LNA Array (v.18.0) (Exiqon) according to array manual. The total 25 μL mixture from Hy3™-labeled samples with 25 μL hybridization buffer were first denatured for 2 min at 95°C, incubated on ice for 2 min. Then hybridized to the microarray for 16–20 h at 56°C in a 12-Bay Hybridization Systems (Hybridization System-Nimblegen Systems, Inc., Madison, WI, USA). Following hybridization, the slides were achieved, washed several times using Wash buffer kit (Exiqon). Then the slides were scanned using the Axon GenePix 4000B microarray scanner (Axon Instruments, Foster City, CA).

### Data processing and analysis

Scanned images were then imported into GenePix Pro 6.0 software (Axon) for grid alignment and data extraction. Replicated miRNAs were averaged and miRNAs that intensities>=30 in all samples were chosen for calculating normalization factor. Expressed data were normalized using the Median normalization. After normalization, significant differentially expressed miRNAs between two groups were identified through Fold change and P-value. Differentially expressed miRNAs between two samples were filtered through Fold change. Finally, hierarchical clustering was performed to show distinguishable miRNA expression profiling among samples.

### Validation with quantitative real-time PCR (RT-qPCR)

To validate microarray data, we analyzed the expression of miRNAs using quantitative Real Time-Polymerase Chain Reaction (RT-qPCR). We used the miScript PCR System (Qiagen) for reverse transcription and RT-qPCR. RNA was converted into cDNA using the miScript II Reverse Transcription Kit and the HiSpec Buffer according to the manufacturers´ protocol. The RT-qPCR was performed with the miScript SYBR® Green PCR Kit in a total volume of 20 μL per reaction containing 1 μL diluted cDNA according to the manufacturers´ protocol. MiR-191-5p served as endogenous control since this miRNA has been reported as a suitable normalizes of miRNA values in plasma.

### Statistical analysis

The comparison of different miRNA expression in plasma between primary resistant patients and sensitive patients was analyzed using the Students t-test. The Fisher’s test was used to analyze the significance of the genetic networks identified by the IPA tool. A p value < 0.05 was considered statistically significant.

### Network and gene ontology analyses

Genetic networks and functional classification of differentially expressed miRNAs were investigated with IPA (Ingenuity Systems, Mountain View, CA), a web delivered tool that enables the discovery, visualization, and exploration of molecular interaction networks in gene expression data.
